# Extending Burk Dehority’s Perspectives on the Role of Ciliate Protozoa in the Rumen

**DOI:** 10.3389/fmicb.2020.00123

**Published:** 2020-02-28

**Authors:** Jeffrey L. Firkins, Zhongtang Yu, Tansol Park, Johanna E. Plank

**Affiliations:** Department of Animal Sciences, The Ohio State University, Columbus, OH, United States

**Keywords:** rumen protozoa, protozoal taxonomy, rumen protozoal monoculture, isotrichid, entodiniomorphid, rumen protozoal 18S rRNA genes

## Abstract

Dr. Burk Dehority was an international expert on the classification and monoculture of ruminal ciliated protozoa. We have summarized many of the advancements in knowledge from his work but also in his scientific way of thinking about interactions of ruminal ciliates with the entire rumen microbial community and animal host. As a dedication to his legacy, an electronic library of high-resolution images and video footage catalogs numerous species and techniques involved in taxonomy, isolation, culture, and ecological assessment of ruminal ciliate species and communities. Considerable promise remains to adapt these landmark approaches to harness eukaryotic cell signaling technology with genomics and transcriptomics to assess cellular mechanisms regulating growth and responsiveness to ruminal environmental conditions. These technologies can be adapted to study how protozoa interact (both antagonism and mutualism) within the entire ruminal microbiota. Thus, advancements and limitations in approaches used are highlighted such that future research questions can be posed to study rumen protozoal contribution to ruminant nutrition and productivity.

## Introduction

Rumen ciliated protozoa enhance methanogenesis (Newbold et al., 2015) and contribute to proteolysis and particularly to intra-ruminal recycling of microbial protein (Hartinger et al., 2018; Firkins and Mackie, 2020). These negative aspects are countered by benefits in limiting the post-prandial depression in ruminal pH through engulfing starch and metabolizing lactate, and some ciliates contribute significantly to fiber degradation (Newbold et al., 2015). Hence, untargeted protozoal suppression can lead to repercussions in the complex ruminal ecosystem and variable efficacy in suppression of methanogenesis (Hristov et al., 2013) and probably for suppression of intra-ruminal recycling to limit nitrogen excretion (Firkins and Mackie, 2020).

The classical ecology of ruminal ciliates was described decades ago (Williams and Coleman, 1992). The rumen ciliates remain a partial enigma today with respect to weighing their pros vs. cons, although they now are less enigmatic thanks to the nearly six decades of pioneering research by our colleague, Dr. Burk Dehority. He was an avid swimmer for decades. Hence, the 2013 symposium in his honor at the joint American Society of Animal Science/American Dairy Science Association meeting in Indianapolis, IN was aptly entitled: “Swimming in the Rumen with Burk Dehority.” What made him a good “rumen swimmer” was his broad thinking. For example, when invited to talk on the role of protozoa in the rumen, he once excerpted part of that title for his main theme that, rather than defining their role, he focused on “protozoa are in the rumen because they can be.” When researchers focus excessively on defining their role from the top–down (teleological vantage), he questioned if they missed opportunities to actually better understand their role from the bottom up (biologically).

Burk Dehority helped shape our own views that ruminal ciliates are in the rumen “because they can be.” For example, should we continue to study inhibiting agents without studying how the protozoal community adapts to them (and how they do it)? Indeed, *Entodinium caudatum* expressed eukaryotic stress response pathways when the inhibitors monensin and wortmannin were introduced (Wang et al., 2020). We questioned if protozoal autolysis in the rumen is less than that projected from cultures (Diaz et al., 2014). We also questioned if ruminal ciliates can be cultured without prokaryotes (Park et al., 2017; Park and Yu, 2018a). Of course, protozoal cultures have led to critical advances in our understanding of the growth rate and function of various species, but Dr. Dehority always conditioned observations made *in vitro*. For example, were increased protozoal numbers in monocultures a direct stimulation by the treatment imposed or a result of indirect inhibition of the uncharacterized prokaryotes in co-culture? Hence, there are some important similarities in prokaryotic diversity in ciliate monocultures compared with *in vivo*, but there also are major differences (Park and Yu, 2018b). Dehority (2003) contextualized the difference between protozoal counts (cells per milliliter) and protozoal pool size (total cells) in the rumen, their ruminal passage or lack thereof (sequestration), and their adaptation to host and diet based on function and taxonomy from the bottom up. Unfortunately, protozoal pool size has rarely been measured (see later discussion).

Dr. Dehority’s scientific passion intersected with his artistic side; his drawings of rumen protozoa led to more sophisticated images in his books, which were the basis for the corresponding author’s (JLF) rumen microbiology class (and used in other classes across different universities). For the current perspective, these images seeded the image database as a lasting legacy to our colleague. Readers are referred to the Acknowledgments section to access this image library. In addition to his artistic talents, his scholarship on the study of ruminal ciliates was also a passion few got to witness; even after retirement, stretching out maintenance procedures to avoid tending them over weekends (Dehority, 2008) was not a reflection of the joy he derived from tending his cultures daily—these revisions were for others to follow. Rather than accepting the standard explanation, he always sought to test hypotheses—typically in elegantly simple ways such as incubating protozoa in the rumen within an apparatus that prevented entry or exit of protozoa across the screen (Ankrah et al., 1990). He successfully defaunated animals but did not rely on defaunation as the main means of explaining a role based on the “because they can” philosophy; he always questioned how the faunation vs. defaunation comparison removed the positive and negative interactions of protozoa with bacteria and fungi.

Our current objective is to review his work and connect his findings with current and especially future research objectives to further our understanding of interactions among protozoa and other microbes in the ruminal pool from a microbiology-nutrition interface.

## Landmark Findings on Ciliated Protozoa

### Swimming in the Rumen

Dehority (1970) described *Buetschlia parva* (family *Buetschliidae*, order *Entodiniomorphida*) in his first PubMed-indexed paper on protozoa in January of 1970. The family *Ophryoscolecidae* is diverse and has the major role with respect to how ciliates influence rumen function by most ruminants fed most production diets. Ophryoscolecids primarily have cilia only near their oral region, although some have other patches (Dehority, 2003). We will maintain the term ‘entodiniomorphid’ to describe this group. The order *Vestibuliferida* has two main families typically identified in the rumen, with the predominant one being *Isotrichidae* (Cedrola et al., 2015). We will maintain the term ‘isotrichid’ to represent this main group, which is fully ciliated; however, we abandon the previous term ‘holotrichs’ because cilial pattern and location does not taxonomically distinguish minor members such as the *Buetschliidae*.

A few months after his first paper, Dehority and Purser (1970) described factors influencing isotrichid counts in the sheep rumen. Dr. Purser and his colleague, Dr. Moir, had important landmark papers on ruminal ciliates from the 1960s. This particular paper was the first among many of Dr. Dehority’s fruitful ventures with international scientists (in this case, Australia). Purser (1961) noted: “Several outstanding biochemical differences between Oligotrichs [i.e., entodiniomorphids] and Holotrichs [i.e., isotrichids] have now been established *in vitro*. However, *in vitro* work can delineate biochemical capabilities only. It cannot predict with certainty the activity of these organisms *in vivo.*” This theme guided Dr. Dehority’s thinking so that even his culture-based studies were designed to reflect important *in vivo* responses.

Only a relatively few experts were truly accepted internationally to verify a new protozoal species or differentiate some closely related species based on morphological features. Therefore, numerous scientists across the globe traveled to the Dehority laboratory or invited him to theirs, and he has coauthored numerous indexed papers with multiple authors using multiple animals from multiple continents. He studied rumen protozoa in relation to feed intake, forage:concentrate ratio, feeding frequency, ruminal pH (Dehority, 2003), and even osmolality (Dehority and Males, 1974). Toward the end of his career, he helped establish protozoal adaptations to body temperature differences between Australian macropods and ruminants (Dehority and Wright, 2014). Based on this work, one of our final exchanges with Burk was when he suggested to briefly decrease the temperature to eliminate protozoa from continuous cultures. Curiously, our continuous culture conditions sometimes greatly increased the relative abundance of *Charonina* (Wenner, 2016), which is often overlooked during typical counting and therefore might contribute more to protozoal ecology than currently known (Wenner et al., 2018). Dehority and Mattos (1978) noted that this genus had an ecology more closely resembling that of entodiniomorphids even though it was then classified with the isotrichids, perhaps envisaging its reclassification in *Entodiniomorphida* (Cedrola et al., 2015). Discussing flagellated protozoa in the rumen and distinguishing a myriad of ciliate species in numerous herbivores across many continents is beyond our current scope, his international legacy can be readily appreciated by searching his indexed publications.

### Microscopic Characterization of Protozoa

Dr. Dehority was particularly adept at culturing bacteria, protozoa, and fungi using a gassing station and characterizing protozoa based on their morphology. Originating with notes shared with colleagues and progressing to a widely used laboratory manual (Dehority, 1993), he was then requested to turn his laboratory-based class and his artfully drawn illustrations into his book (Dehority, 2003). In both resources, Dr. Dehority explained how to effectively count protozoa with both high accuracy and precision.

Size is a relative distinction among species, but relatively little gain in knowledge has been made despite the long-held concept that generic or even species distinctions do not explain changes in protozoal biomass or activity (Whitelaw et al., 1984). Although not well-understood in the literature, he emphasized the concept that increasing grain inclusion in a diet increases protozoal cell density by a combination of more substrate to stimulate cell growth but also a lower ruminal fluid volume to concentrate those cells (Dehority, 2003). He reasoned that cell counts should be multiplied by ruminal volume to derive a rumen pool. The nutritional need to move from cell counts to ruminal and duodenal biomass led to our development of a qPCR technique that relies on collecting a protozoal standard (Sylvester et al., 2005) and our development of a videographic technique (Wenner et al., 2018) to adapt counts to volume. Both counts and protozoal 18S rRNA gene copies are useful (Kittelmann et al., 2015), but both also have pros and cons that will be described in a subsequent section.

Speciation of protozoa based on visual morphology is challenging. An excellent example is the case when Dehority (1994) exhaustively characterized and offered a biological explanation why different species should be collapsed into differing morphological types of the same species, *Entodinium dubardi*. Prior to the Dehority symposium, the corresponding author (JLF) asked him what prompted this paper, and his reply was “sheer frustration.” This response was from a scientist who described and named 21 new species and had at least two species named after him. This textbook case documents why some morphological characteristics are stronger indicators of taxonomic distinction of species than others. *Entodinium caudatum* has three main forms of spination (from none to very long), assuming spination is a defense against predation by larger protozoa and is not needed in monocultures (Dehority, 2003). Similar changes in morphology were demonstrated with extended culture of *Ophryoscolex* (Miltko et al., 2006).

### Visual Library

Based on Dr. Dehority’s tutelage, many researchers classified protozoa into the genera *Isotricha*, *Dasytricha*, *Entodinium*, and the subfamilies *Diplodiniinae* and *Ophryoscolecinae* (with further classification of the genera *Ophryoscolex* and *Epidinium*). However, as Burk’s health declined, we prioritized our goal of creating a photographic library of a broad range of protozoal species to leave a lasting framework for future researchers (see Acknowledgments for directions to access the image library). Burk agreed to serve as a collaborator in the fall of 2014, when one of the coauthors (JEP) began to collect rumen fluid samples to supplement existing images of confirmed species. *Dasytricha ruminantium* images and *Isotricha intestinalis* videos were added. As discussed previously, Burk distinguished *Charonina ventriculi*, and images are now better represented in the library to help future researchers distinguish this atypical protozoan’s unusual features (Wenner et al., 2018).

Collecting a sample and preparing the specimen for proper resolution requires attention to detail, patience, and experience—especially for some genera or species that are very small (such as *C. ventriculi*) or for which morphology types are very similar (and might require a high-resolution microscope). Iodine staining highlights skeletal plates, a morphological property differentiating species in the *Ophryoscolecinae* subfamily. To avoid the obfuscation from staining both skeletal plates and intracellular starch or glycogen, Burk recommended sampling from donor cows fed an all forage or high fiber diet or withholding feed for 24 h prior to collection. Due to ongoing research projects in our dairy herd (presumably a limitation for numerous researchers), these suggestions could not be followed. Collection before feeding might seem a disadvantage for taxonomic approaches based on morphological characteristics compared with molecular approaches (discussed subsequently), but we also note that large particles also typically are screened even when DNA is extracted from rumen contents containing particulates. Our best option was to sample immediately prior to the morning feeding (cows were fed twice daily but eating at *ad libitum*). Flocculation and aspiration of the top layer reduced the concentration of particulates that also stained with iodine, but we also noted that large protozoa were sometimes inadvertently removed from the sample. During this process, we captured images that were confirmed by Dr. Dehority to be *Ostracodinium gracile* and *O. obtusum*.

Aware of our challenges to find rare images, Burk generously offered to donate additional materials. Electronic presentations entitled ‘Classification and Morphology,’ ‘Counting Methods,’ ‘Species Identification,’ and ‘Difficulties with *Entodinium*’ are insightful and included in the image library. Burk was helping us replace a lost monoculture of *Epidinium caudatum* that had failed to revive after extended cryopreservation (in which he also was experienced through collaborations with others). Two of the coauthors (JEP and TP) collected fluid from a rumen-cannulated cow in Columbus, Ohio that was previously confirmed to have moderate abundance of *Ep. caudatum*. Unfortunately, due to the then low concentrations of *Epidinium*, no monocultures were confirmed. Despite declining health, Burk found a more promising donor in Wooster, OH, United States. He isolated and confirmed a clone culture for our subsequent usage. As was his decades-old practice, he invited two coauthors (JEP and TP) to Wooster to isolate a single cell for clone culture, leading to one of the videos in the visual library.

On January 15, 2016, one of the coauthors (JEP) emailed Burk a short video clip and a few still photographs of a specimen previously undocumented by our laboratory. On the 18^*th*^, our last exchange, Burk’s return email expressed the enthusiasm that marked his scientific career; although he had cataloged this rare species, it was the first image in our collection confirmed as *Buetschlia parva*. Our final efforts also identified images as *Entodinium rostratum, Ostracodinium trivesiculatum, Epidinium quadricaudatum*, and *Ep. parvicaudatum*. The authors thank Dr. Svetlana Kisidayova (Slovak Academy of Sciences, Košice, Slovakia) and Raul Franzolin (University of São Paulo, Brazil), who graciously identified more protozoa in the unlabeled photographs of our collection that had not yet been classified because of his swift decline and passing on February 9, 2016.

### Outline for Mentoring the Technique for Protozoal Counting

Dr. Dehority’s technique is captured in a PowerPoint presentation in the image library (see Acknowledgments for directions to access the link), with some of our own examples from his mentoring highlighted below.

(1) Pass ruminal samples only through one or two layers of cheesecloth to remove large particles of feed before staining (for good viewing). Larger protozoa can be entrapped and under-represented using multiple (i.e., more than 2) layers (Dehority, 1984). He demonstrated and patiently evaluated sampling and subsampling approaches, and use of a glass open-bore pipette was especially critiqued by Dr. Dehority. We note that, aspiration of flocculated particles can help remove contamination, as described above, but this process could differentially remove some of the larger protozoal species.
(2) Researchers should first learn to differentially count *Isotricha*, *Dasytricha*, *Epidinium*, *Ophryoscolex*, *Entodinium*, and subfamily *Diplodiniinae*. More details and practice are needed to distinguish the subfamilies *Entodiniinae* (which includes only *Entodinium*), *Diplodiniinae*, and *Ophryoscolecinae* (Dehority, 2003). For greater depth, readers are referred to Dr. Dehority’s PowerPoint on distinguishing *Entodinium* in the image library.
(3) Either unstained or stained samples, as described in his manual (Dehority, 1993), researchers should focus up and down the cilial patterns to distinguish *Isotricha* (longitudinal patterning) from *Dasytricha* (cilia spiraling around the cell); *I. prostoma* is distinguished from *I. intestinalis* by the vestibule. If using iodine, visualize as soon as possible, and its concentration might need to vary based on the sample (especially if varying in starch).
(4) Genera can be further distinguished based on cilial zone patterns, the number of contractile vacuoles, the number and shape of skeletal plates, and the shape and location of the macronucleus. However, sometimes smaller *Diplodiniinae* can be distinguished using methylene blue or iodine staining only at a higher magnification on separate slides with cover slips; their percentage can subsequently be multiplied by a normalizing count from a matched subsample.
(5) Caudal spine location has some value in taxonomy, but spines should not be used in length measurement, and spination can vary with culture conditions of some entodinia (see above discussion).

### Clone Cultures of Entodiniomorphids

Monocultures made important contributions to ruminal protozoology since the 1950s, but Dr. Dehority established numerous conditions and improvements that standardized approaches used to define the roles of substrate source, how the substrates were processed, and deviations of temperature (Fondevila and Dehority, 2001a; Dehority, 2010); he also verified inhibition by decreasing pH (Dehority, 2005). Protozoal cultures typically are fed daily but can be maintained over weekends without feeding (Dehority, 2008). In contrast, the generation time (the typical term Burk used to represent protozoal doubling time) of several species studied, including large and slower growing strains, can be shortened from > 2 days to approximately 12 h by shortening transfer interval (Dehority, 1998, 2004). This relationship appears to hold over different protozoal species such that the larger ciliates occupy a similar mass in the culture tube even if proportionately lower in numbers (Sylvester et al., 2009; Dehority, 2010).

The competition between protozoa and bacteria for substrate has led to standardized methods (Dehority, 2008) that help to maintain higher counts in monocultures. However, protozoa can grow rapidly on a mass basis when one considers that numerous generations of co-cultured bacteria would be needed to regenerate the same amount of biomass in a single dividing protozoan. Even the numbers of bacteria derived from most-probable number, which typically underestimates true abundance of bacteria (Firkins and Yu, 2006), were > 10^3^ the counts of protozoa in the ‘protozoal’ monocultures (Dehority, 2008). Although not expressly measured, Burk commented that prokaryotes make a significant contribution to the total microbial biomass. Despite the apparent competition by prokaryotes and protozoa for the same substrate, protozoal cultures grew best with live rather than dead (autoclaved) bacteria (Fondevila and Dehority, 2001a), which might be a result of continuous recruitment of endosymbionts, as discussed later.

One of the authors (JLF) questioned Dr. Dehority if protozoa overshooting growth in culture can lead to subsequent lysis if substrate or growth factors are subsequently depleted. This discussion shifted our thinking that slowing growth rate “because they can” might maintain a competitive advantage against bacteria if feed intake and ruminal passage rate are low (Firkins et al., 2007). Based on principles derived from the non-rumen ciliate, *Paramecium* (Berger, 2001), we reasoned that an upshift in protozoal growth rate associated with increasing nutrient supply can be countered by a downshift in growth rate with decreasing nutrient supply as mediated by an arrested eukaryotic cell cycle.

Protozoal cultures adapt to monensin after a few transfers (Sylvester et al., 2009). After abrupt introduction to monensin, protozoal growth was apparently stunted (as assessed by protozoal 18S rDNA copies per unit of total nucleic acids) similarly to the effects of not feeding. Even after longer adaptation, monensin increased the generation time of mixed protozoa by 5 to 6 h in continuous culture (Ye et al., 2018). Yet, protozoal abundance was not diminished when monensin was fed to dairy cattle (Oelker et al., 2009; Reveneau et al., 2012), supporting our supposition that generation time being shorter than retention time is not the only factor that limits the abundance of protozoa in the rumen. Decades prior, Potter and Dehority (1973) reasoned that decreasing feed intake decreases the passage rate of ruminal fluid and diminishes its role in affecting protozoal generation time. At that time, attachment to feed particles and therefore passage with the particulate phase was not yet emphasized.

Although entodiniomorphid adhesion to fiber particles was assumed based on observed attachment by *Epidinium* (Bauchop and Clarke, 1976), the attachment is passive and weak except when associated with feeding (Jouany and Ushida, 1999). Our experience has been that *Epidinium* counts vary considerably among cows (Sylvester et al., 2005) even though this genus was curiously resistant to toxicity by coconut oil (Reveneau et al., 2012). The intimate association with plant matter was not exhibited in all strains of *Ep. caudatum* monocultures (Dehority, 2010), and the mechanical disruption of plant material that can be visualized microscopically (and as described as ‘shredding’ in conversations with Burk) for these strains of *Epidinium* should not be extrapolated to all of the entodiniomorphids, anyway (Jouany and Ushida, 1999). Isotricha has a specialized ability to attach to feed particles and the ventral reticulorumen wall (Jouany and Ushida, 1999). However, based on their ecology (below), attachment would seem more important for sequestration in the ventral rumen than for gaining access to feed particles.

## Ecological Differences Between Entodiniomorphids and Isotrichids

### Isotrichid Ecology

That the isotrichids were fully ciliated and had a different ecology than the entodiniomorphids has been long known (Purser, 1961). They typically establish after the entodiniomorphids in young ruminants (Yáñez-Ruiz et al., 2015) even after inoculation from a fully faunated donor (Cersosimo et al., 2019). Isotrichids become opaque after rapidly converting sugars and very small starch granules into glycogen, and their increased density causes them to sink to the ventral reticulorumen (Dehority, 2003). Diaz et al. (2014) argued that sinking would be facilitated by intentional swimming behavior to pass ventrally through the fibrous rumen mat and even by contorting their cells to move around particulates. Concentrating ventrally below the reticulo-omasal orifice allows isotrichids to evade passage and explains their lower numbers recovered from samples collected near a ruminal cannula (i.e., they migrated to the ventral rumen and reticulum) within a few hours post-feeding. The more feedings per day, the more cyclical patterns in isotrichid counts as measured from these dorsal sampling locations (Abe et al., 1981). However, a pivotal study (Dehority and Tirabasso, 1989) started with a simple but novel question: what would happen to isotrichid counts if the animal was not fed at its regular interval? The isotrichid counts in the ruminal samples (near the cannula) peaked right on schedule coinciding with their ‘trained’ feeding pattern even when the animals were not fed. Migratory behavior to swim dorsally seemingly would have to precede chemotaxis toward an increasing gradient of nutrients in the dorsal area. Dr. Dehority reasoned that chemotaxis would be reduced when isotrichids were repleted with glycogen; however, with glycogen-depletion, chemotaxis would be enhanced by some signaling mechanism coinciding with dorsal migration.

Dr. Dehority was never able to maintain long-term cultures of the isotrichids (genera *Isotricha* and *Dasytricha*), although he cited the importance of controlling excess glycogenesis as one of the factors for culturing *Dasytricha* (Dehority, 2003). Diaz et al. (2014) hypothesized that increased glycogen storage capacity decreased isotrichid chemotaxis. Although this supposition remains unconfirmed, improved glycogen quantification methods (Hall, 2019) could be integrated with cell signaling measurements (later section) to better understand cellular mechanisms underpinning their ecology.

Although generation times have not been well-studied in isotrichids compared with entodiniomorphids, hydrogenosomal function and aerotolerance have been better described for isotrichids (Williams and Coleman, 1992). Oxygen enters the rumen via feed and water (i.e., near the rumen mat) and by diffusion from the blood across the rumen wall (where they sequester); consequently, electron transport-linked ATP generation from ingested glucose and stored glycogen would be enhanced if they can use O_2_ as a terminal electron acceptor (Williams and Coleman, 1992). Although hydrogenosomes have been linked with O_2_ consumption (Newbold et al., 2015), to our knowledge, the actual mechanism of O_2_ usage has not been clarified since it was summarized by Williams and Coleman (1992). Further study is needed to distinguish hydrogenosomal synthesis of ATP via succinyl CoA, which could not be verified using biochemical techniques (Williams and Coleman, 1992) but was predicted using metatranscriptomics (Qi et al., 2011). Extra ATP yield could offset extra ATP usage for glycogenesis of ingested sugars and potential glycogen cycling (Teixeira et al., 2017). Williams and Coleman (1992) noted glycogen cycling in isotrichids but also suggested that isotrichids collect near the reticulorumen wall to metabolize glycogen with greater efficiency of ATP production through O_2_ respiration. This migration away from sugars leaching from freshly ingested feed also could improve energetic efficiency by decreasing glycogen cycling.

After discussion with Dr. Dehority, we questioned if the high expected protozoal autolysis (Dijkstra and Tamminga, 1995) is inflated as a result of necessarily relying on measurements that were derived *in vitro*. In this case, Prins and Van Hoven (1977) dosed a relatively large amount of substrate to a previously starved *Isotricha* monoculture at the same time as antibiotic was introduced, thus likely abruptly inhibiting lactilytic bacteria in co-culture. Accumulation of lactate (pH sometimes < 5.0) in that study would not reflect normal conditions in monoculture, let alone *in vivo*. The diurnal rhythm of migration and subsequent sedimentation might be heavily entrenched in isotrichid ecology. Hence, Diaz et al. (2014) postulated that extensive autolysis by isotrichids in culture tubes could result from their inability to swim away from lytic conditions as would occur when they migrate to and sequester in the ventral rumen.

### Acquisition of Substrate

Cilial pattern and coordination in beating pattern are intricately linked to the ecology of isotrichids. They rapidly consume glucose, fructose, and sucrose and rapidly store glycogen; however, they do not appear to be capable of using lactose (Dehority, 2003). They also can produce lactate (Williams and Coleman, 1992), as noted above. Those latter authors documented swimming behavior and chemotaxis to soluble nutrients. However, Diaz et al. (2014) explained how their swimming behavior, including rotation like a screw and mechanical contortion, allows them to move around obstacles as they migrate between the rumen wall and mat. Isotrichids are highly chemotactic toward glucose and xylose (Diaz et al., 2014) and almost certainly other sugars. Peptides can be both chemoattractive and chemorepellent to isotrichids (Diaz et al., 2014; Roman-Garcia et al., 2019). Hence, uncontrolled glycogenesis *in vitro* (Jouany and Ushida, 1999; Hall, 2011) probably is lessened *in vivo* because of much less abrupt increase in sugar availability. Williams and Coleman (1992) also refuted excess sugar uptake and glycogenesis as a mechanism for isotrichid autolysis in favor of toxicity resulting from a buildup of acidic endproducts *in vitro*.

In contrast to the isotrichids, the entodiniomorphids ingest fibrous particles and larger starch granules and appear to be important lactate consumers, not producers (Williams and Coleman, 1992; Jouany and Ushida, 1999). Ingestion of particles through the vestibulum using specialized cilia has been described (Jouany and Ushida, 1999). The adoral cilial zone of entodiniomorphids probably helps both with ingestion and locomotion (Dehority, 2003). Although less responsive than isotrichids, the entodiniomorphids exhibited chemotaxis toward glucose, xylose, and peptides (Diaz et al., 2014). Those authors presented a model in which entodiniomorphids maintain moderate but constant chemotaxis toward soluble nutrients leaching from freshly ingested or rapidly degrading plant particles. Consistent chemotaxis should maintain the entodiniomorphids swimming freely in fluid but without long-term attachment while passing with the particulate phase (see earlier discussion). Association with particles should lessen rate of protozoal outflow that would otherwise occur with the faster passage of fluid (Orpin, 1985). Also, entodiniomorphids probably are more bacterivorous and proteolytic than isotrichids (Newbold et al., 2015; Firkins and Mackie, 2020), and the majority of bacteria are in the particulate phase (Sok et al., 2017).

### Cell Signaling

Like other eukaryotic organisms, ruminal ciliated protozoa presumably can respond to external and internal factors in a regulated manner to control their cell cycle, and signal transduction is essential to these responses (Firkins et al., 2007). Although well-described for the environmental ciliate models *Paramecium tetraurelia*, *Paramecium multimicronucleatum*, and *Tetrahymena thermophila* (Plattner, 2017), signal transduction in rumen protozoa was first indirectly demonstrated using wortmannin [an inhibitor of phosphoinositide 3-kinase (PI3K)], insulin (a growth factor), genistein (inhibitors of receptor tyrosine kinase), and U73122 (an inhibitor of phospholipase C) using a chemotaxis assay and quantifying engulfment of fluorescent beads that mimic bacteria in size and surface charge (Diaz et al., 2014). Trafficking of vesicles typically involves second messengers such as Ca^++^ and specific phosphoinositides.

Understanding of cellular signaling by ruminal ciliates was expanded using commonly available eukaryotic inhibitors and activators. The PI3K inhibitor, wortmannin, depressed chemotaxis in isotrichids but increased chemotaxis to glucose in entodiniomorphids (Diaz et al., 2014). Protein kinase G activation by cyclic GMP (based on the nitric oxide stimulator, sodium nitroprusside) was projected to activate chemotactic directional turning for entodiniomorphids but not for isotrichids. Roman-Garcia et al. (2019) suggested that increased concentration of NO${}_{3}^{2-}$ disrupted protein kinase G-stimulated chemotaxis by entodiniomorphids to peptides but not to glucose. Cellular receptors and the associated signaling mechanisms need much more research attention in ruminal ciliates.

In the transcriptome of *En. caudatum*, at least 25 different putative signal transduction pathways were recorded (Wang et al., 2020). Those transcripts relatively highly expressed included well-described eukaryotic pathways MAPK, Ras, calcium, cGMP-PKG, cAMP, FoxO, phosphatidylinositol, sphingolipid, TOR, PI3K-Akt, AMPK, Wnt, and Apelin. The expression of these pathways reflects the ability of *En. caudatum* to regulate its transcription, translation, ribosome biogenesis, cell growth, proliferation and differentiation, cytoskeletal organization and dynamism, chemotaxis, metabolism, secretion, calcium homeostasis, cell fate, gene transcription, apoptosis, cell-cycle control, oxidative stress resistance, etc. The functionality of these signal transduction pathways may also play an important role in their fitness and overall contribution to rumen function. Key signaling differences between isotrichids and entodiniomorphids need further verification using more specific techniques such as specific antibodies, which should become more feasible with whole genome sequencing (Park et al., 2018).

## Quantifying Protozoal Contributions to Supply of Protein

### Ruminal Pool Size and Passage Rate: From Culture to Cow

Turnover of the ruminal protozoal pool is the net of growth relative to passage and recycling. Growth involves primarily cytokinesis associated with mitosis, although conjugation (meiosis) is sporadically observed in ruminal ciliates (Williams and Coleman, 1992). Little progress has been made on the molecular events regulating growth of ruminal ciliates, although complexity can be expected based on extrapolation from non-rumen ciliate models (Wang et al., 2017). Clearly, the relatively rich source of nutrients and relatively fast passage rate from the rumen would be unique for ruminal ciliates compared with such conditions influencing environmental ciliates. These conditions also support horizontal transfer of prokaryotic genes into protozoa (Newbold et al., 2015).

Dr. Dehority emphasized that generation time (net of cell growth and lysis) was highly related to transfer interval (Dehority, 1998, 2004, 2008). At each transfer, an abrupt halving of cell numbers would coincide with fresh substrate, and growth would respond quickly and as coordinated with decreasing transfer interval until a minimum generation time could be derived. With faster growth, we would expect a larger percentage of dividing forms and more nucleic acid and N per cell; even so, the observed percentage of dividing forms was not always clearly related to transfer interval (Sylvester et al., 2009). The ciliate’s cellular signaling mechanisms should be coordinated with its cell cycle (Firkins et al., 2007; Diaz et al., 2014), which is supported by transcriptomics profiling in *En. caudatum* (Wang et al., 2020). These same eukaryotic cell cycle controls should both increase growth and decrease growth rate, depending on stage of incubation. At 30 h after the previous feeding (i.e., 6 h past the scheduled 24-h feeding that was interrupted), protozoal cultures already started losing cell numbers (Sylvester et al., 2009); abrupt changes in feeding pattern presumably precluded a downshift in cell cycle control that was not quick enough to avoid autolysis.

Faster passage rate in the rumen is typically associated with increasing feed intake, which stimulates protozoa to replenish their numbers but also coincides with increased substrate supply. Potter and Dehority (1973) noted that ruminal turnover rate (which is positively correlated with feed intake) was suggested to be the dominant factor associated with protozoal counts when feed intake was high. Czerkawski (1987) suggested that the mean residence time for protozoa would be approximated by the retention time of the particulate phase in the rumen. Measurement of omasal outflow of cells avoids the destruction by the acidic abomasum and, combined with ruminal pool size of cells, allows quantification of generation time of total and individual taxa (Karnati et al., 2007). Those authors noted that protozoal generation time approximated the ruminal retention time of Yb-labeled forage. A model parameterized based on cell counts emphasized loss of cells in the omasum (Hook et al., 2017). However, cell counts in the omasum should be expected to be lower than in the rumen (Czerkawski, 1987) because omasal counts can be diluted by bypass of drinking water that does not mix with the rumen contents and by destruction of omasal cells from abomasal backwash resulting from relaxation of sphincters following euthanization (Firkins and Yu, 2006). Those authors explained that ruminal outflow (rumen volume multiplied by fluid dilution rate) typically overestimates cellular outflow and should be avoided.

Passage of ruminal ciliates needs context to connect prior expectations with more current thinking. First, we argue that, unlike many of those publications and based on numerous discussions with Dr. Dehority, the predominant entodiniomorphids do not sequester in the rumen as do the isotrichids. Some of the expectation for sequestration by entodiniomorphids is based on interpretation of ‘attachment.’ In contrast with intimate and long-term attachment by other important ruminal microbes such as by cellulolytic bacteria, Dehority (2010) defined attachment as being “closely associated with the insoluble particulate matter.” Second, protozoa traditionally have been expected to be about 50% of the microbial biomass in the rumen and even as high as 70% (Jouany, 1996). This high a pool size is inconsistent with the autolyzing argument—how can the protozoal pool size be so high if they have extensive autolysis? In contrast, if the pool size is so high, then autolysis would have to be high to explain the much slower ruminal outflow of protozoa compared with bacteria if the predominant entodiniomorphids do not sequester. We queried this apparent quandary during useful discussions with Burk.

The ruminal pool size of protozoa has rarely been measured at the same time and using the same technique as that used to measure their ruminal outflow (Firkins et al., 2007). Those authors explained why exogenous ^14^C-choline almost certainly overestimated protozoal ruminal pool size yet typically was not used to quantify ruminal outflow of protozoal N. Because of the difficulty in collecting a purified sample of choline, specific activity was instead related to radioactivity of ^14^C per unit of N in samples that were theoretically pure protozoa, whereas those samples were likely significantly contaminated with bacterial N (bacterial cells have virtually no choline). In addition, removal of protozoa for short-term culture needed to enrich them with ^13^C-choline followed by washing likely would introduce stress responses that would increase their generation time. This perturbation likely disturbed the assumption that ^14^C-choline in protozoa turns over the same as unlabeled choline. That is, the ^14^C-choline specific activity would not be diluted as rapidly as it would have without perturbation. These types of studies should be repeated using better procedures such as a quantitative PCR assay (Sylvester et al., 2005). Those authors emphasized the need to assess recovery of 18S rDNA copies just like one would use an internal standard for any chemical assays, whereas few users of this approach seem to be evaluating recovery.

Very early expectation for such high contribution of protozoal biomass (at least 50% of the microbial biomass) were at least in part derived from calculations based on protozoal volume and converted to biomass. However, these prior protozoal volumes derived using the geometric formula for cylinders were probably overestimated by 25 to 40% (Wenner et al., 2018). Based on these arguments, rather than 50%, perhaps a more accurate expectation should be 25% of the microbial biomass being derived from protozoa in the rumen compared with 15% in the ruminal outflow for lactating dairy cattle (Ahvenjärvi et al., 2018). Similar contributions to outflow in dairy cattle (about 17%) were derived using various approaches methods (Sok et al., 2017; Fessenden et al., 2019) that were not based on faulty microbial marker systems (Firkins et al., 1998). More studies are needed to assess factors influencing rumen protozoal N pool size and outflow with animals under production situations.

### Protozoal Contribution to the Host’s Nutrition

The ruminal outflow of protozoa deserves future study because protozoa influence the amino acid profile (especially lysine) of microbial protein (Sok et al., 2017). Moreover, high intake and fast ruminal passage rate presumably increase the efficiency of protozoal protein synthesis just as is expected for bacteria (Firkins et al., 2007). With respect to intra-ruminal N recycling, there is a high flux of rapidly turning over N that equilibrates with ruminal ammonia-N and likely has minimal effect on the efficiency of microbial protein synthesis compared with lysis of microbial protein (Oldick et al., 2000). Lysozyme and protease inhibitors (Park et al., 2019) offer potential to better ascertain mechanism and quantitative estimates of protozoa-mediated degradation of dietary protein and recycling of bacterial protein. Greater intake and faster ruminal passage might lessen intra-ruminal recycling caused by protozoa compared with source data mostly from low producing animals (Firkins et al., 2007). Therefore, protozoal autolysis and predation-derived bacterial lysis need better quantitative estimates to help improve feed efficiency and lessen environmental impact of ruminant enterprises under normal feeding conditions (Firkins and Mackie, 2020).

## Interactions of Protozoa With Other Community Members

### Interactions With Bacteria

Predation based on qualitative (i.e., which bacterial types are preferred prey) and quantitative (i.e., loss of dosed bacterial cells) bases has been summarized by Williams and Coleman (1992) mostly from their own classical studies. When numbers of dosed planktonic bacteria consumed by monocultures of protozoa are extrapolated to *in vivo*, protozoa can theoretically clear the entire ruminal pool of bacteria in just a few hours (Hristov and Jouany, 2005). Those authors also noted that bacterial consumed by protozoa that were collected *in vivo* is less than corresponding bacterial consumption from long-term monocultures. In addition, feeding particulate matter will fill protozoal internal space and limit their bacterial engulfment compared with monocultures that were starved before dosing bacteria without feeding. Those authors concluded that bacterial cell walls are more slowly degraded, so protozoa degraded Gram-positive bacteria more completely compared with Gram-negative bacteria. Hence, significant amounts of partially digested fragments are released into the ruminal fluid and contribute to intra-ruminal N recycling. Firkins and Mackie (2020) emphasized the need for more studies like that performed by Belanche et al. (2012) to assess the importance of protozoal predation under situations that better represent high producing animals.

The findings and limitations of defaunation studies were extensively detailed by Newbold et al. (2015). More evaluation of defaunation perturbations such as length of adaptation time and potential shifts in prokaryotic composition are needed in short-term studies such as that carried out by Morgavi et al. (2012). Relatively few studies have evaluated the effect of defaunation using animals fed at relatively high feed intakes (Firkins et al., 2007). Moreover, some studies must be qualified based on the choice of markers used. For example, those authors explained why diaminopimelic acid (DAP) can be a biased bacterial marker, typically inflating ruminal outflow of bacterial N; because DAP is converted to lysine by protozoa (Martin et al., 1996), this inflation is probably worse in defaunated animals. Discerning readers should discount some reports from which bacterial N flows derived using DAP approached the magnitude of their respective non-ammonia N flows (inferring unreasonably low rumen-undegraded protein).

Quantitative measures often require collection of a respective standard representing the bacterial and protozoal communities. Washing without filtering does a poor job of removing contaminating bacteria (Sylvester et al., 2005). Typically, such cells could be fixed with a low concentration of formaldehyde. However, formaldehyde could disrupt amino acid (especially lysine) profile (Sok et al., 2017). Although multiple washes reduce bacterial contamination, the greater stress likely promotes autophagy and even autolysis at rates greater than would be occurring in undisturbed cells in the rumen (Firkins et al., 2007), so harvesting technique is critical. Less stress from better short-term fractionation (Teixeira et al., 2017) or culturing with a much more limited contribution of bacteria is needed to understand the role of protozoa with less conditional qualification of results.

When considering fresh ruminal isolates or longer term monocultures of protozoa, the co-cultured bacteria in protozoal monocultures do not necessarily have a community structure that would be typical of the rumen (Park and Yu, 2018b). These prokaryotes probably have been naturally selected over time for an ability to survive predation while also withstanding typical feeding intervals of 24 h. Protozoal formation (the main storage form of reserve carbohydrate) is much more extensive than bacterial reserve carbohydrate (Teixeira et al., 2017). Although particularly extensive for isotrichids consuming sugars (earlier discussion), entodiniomorphids also rapidly convert ingested starch to glycogen (Bełżecki et al., 2017). Glycogen accumulation in *En. caudatum* was associated with inhibition by antibiotics (Park et al., 2017). Glycogenesis was probably an indicator, not a cause. For these reasons, in addition to prior discussion on the benefit of protozoal glycogen formation to limit low pH troughs, future research on glycogen regulation is recommended.

Protozoal communities fall into distinctive categories probably because of antagonism among species (Kittelmann et al., 2016). Protozoal community structure is important also for bacterial community composition because of the potential for selective predation of bacteria by different protozoal genera (Park and Yu, 2018b). Even so, researchers need to consider the difference between direct predation (i.e., assuming bacteria are the target) and indirect ‘grazing’ (i.e., bacteria that adhere to engulfed particulate matter). None of these concepts has been well-studied in the rumen other than by *post hoc* association. For example, bacteria were visualized to collect after advancing stage of fibrous particle degradation (Bohatier et al., 1990). Hence, protozoal predation and contribution to intra-ruminal N recycling deserve further attention but with a more holistic perspective (Firkins and Mackie, 2020). Researchers need to prevent oversimplification of results because of the complexity of microbial community webs. We still have poor representation of ruminal bacteria, particularly some clades of the Bacteroidetes, so functionality of these phylogenetic associations needs further attention (Firkins and Yu, 2015).

### Interactions With Fungi and Archaea

Like bacteria, fungi compete with protozoa for substrate but also need to avoid predation of their zoospores (Edwards et al., 2017). Those authors described the high activity of rumen fungi against recalcitrant fiber. Thus, one might expect defaunation (removal of protozoa) to increase fiber digestibility if fungi expand into the void resulting from defaunation. In contrast, defaunation was also associated with a decrease in anaerobic fungal counts and the 16S rDNA copies of some important cellulolytic bacteria, suggesting an overall benefit to the entire fibrolytic consortium for improved NDF digestibility associated with presence of protozoa (Newbold et al., 2015). Dehority and Tirabasso (2000) determined that the competition for substrate by bacteria might be enhanced by bacterial antibiosis (presumably a heat- and protease-stable agent) against fungi. Dr. Dehority was involved in research explaining how bacteriocins target other bacteria (Chan and Dehority, 1999), which could also be affected by the protozoal community if they have a major role shaping the bacterial community. Bacteriocin research leaves unanswered questions for animal production (Firkins, 2010).

Because of interspecies H_2_ transfer from protozoa, fungi, and some bacteria to hydrogenotrophic archaea, further research is needed to explain why protozoal inhibition was not recommended as a CH_4_ mitigation strategy (Hristov et al., 2013). Many of the methanogens are extracellular and therefore are not specific to H_2_ source. Moreover, not all ruminal protozoa have hydrogenosomes (Hackstein and Tielens, 2010). For example, *En. caudatum* lacks a hydrogenosome (Park et al., 2017) and does not consistently shift fermentation toward butyrate as shown with mixing monofaunated cultures (Zeitz et al., 2013). Defaunation *in vivo* is typically associated with a decrease in molar ratio of butyrate (Newbold et al., 2015). *En. caudatum* still expresses hydrogenases (Wang et al., 2020), whereas hydrogenosome-linked hydrogenases in other protozoa might be more constitutively linked with fermentation pathways because some fermentative enzymes (especially if sensitive to O_2_) also might be hydrogenosomal, as documented for *Dasytricha* and *Isotricha* (Williams and Coleman, 1992). Although counts of mixed protozoa were positively associated with methanogenesis (Guyader et al., 2014), a decrease in protozoal counts also was associated with a decrease in NDF digestibility and in dry matter intake. Decreased NDF digestibility *per se* should decrease the relative fermentation through acetate or butyrate and thereby also depress methanogenesis. Depressed dry matter intake would require more days on feed for growing ruminants and more animals to maintain milk production, thus eroding the benefit of protozoal suppression strategies from a systems perspective. Variation among ciliates and their associated methanogens, makeup of the carbohydrate, and potential non-additive responses associated with defaunation are complicating factors limiting our understanding of the role of protozoa in methane production (Hristov et al., 2013; Zeitz et al., 2013). Newbold et al. (2015) discussed the differing relationships between protozoal species and methanogens; among those apparent differences, compared with entodiniomorphids, the isotrichids probably support more methanogenesis because of greater O_2_ consumption. Both dissolved concentrations of O_2_ and H_2_ (entry with feed or production, respectively, minus their consumption) influence redox potential, which is associated with and likely influences microbial community and function (Huang et al., 2018).

## Molecular Approaches to Study Protozoal Ecology

### Protozoa-Specific PCR-Based Analysis

The diversity and abundance of rumen ciliates have been examined using morphology- or cultivation-independent molecular approaches, primarily DNA-based and 18S rRNA-targeting methods employing PCR amplification. The early molecular approaches include cloning and sequencing of 18S rRNA gene amplicons (Karnati et al., 2003; Huang and Li, 2018), denaturing gradient gel electrophoresis (DGGE) of such amplicons (Regensbogenova et al., 2004; Sylvester et al., 2005), and quantification of 18S rDNA copies using real-time PCR (Sylvester et al., 2004; Skillman et al., 2006; Saminathan et al., 2017) using protozoa-specific primers. Technically, these molecular approaches circumvent some of the previously described limitations of the morphology-based microscopic identification, including lack of expertise, length of time to count numerous samples, potential misidentification, and morphological variations of the same taxa. In recent years, qPCR for quantification of total protozoa and amplicon sequencing, nearly exclusively with a next-generation sequencing (NGS) technology (primarily MiSeq of Illumina, Inc., San Diego, CA, United States), has been the primary molecular approaches used in compositional and diversity analyses of protozoal communities in the rumen. Protozoa-specific primers are required for accurate and reliable analyses using both approaches.

Several sets of universal rumen protozoal primers have been used to amplify either the nearly full-length or a region of the protozoal 18S rRNA gene. As in the case of prokaryotic analyses based on 16S rRNA gene amplicons, choice of the primers and the hypervariable regions of the marker gene targeted can substantially affect the accuracy and reliability of the community analysis (Bonk et al., 2018). This potential limitation certainly applies to PCR amplicon-based analysis of rumen protozoa. Among the protozoa-specific primers targeting the 18S rRNA genes, most studies used primers RP841F (5′-GACTAGGGATTGGAGTGG-3′) and Reg1302R (5′-AATTGCAAAGATCTATCCC-3′) targeting the V5–V8 regions (Regensbogenova et al., 2004; Kittelmann and Janssen, 2011) and P-SSU-316F (5′-GCTTTCGWTGGTAGTGTATT-3′) and GIC758R (5′-CAACTGTCTCTATKAAYCG-3′) covering two signature regions of rumen ciliates (Sylvester et al., 2004; Ishaq and Wright, 2014). The RP841F/Reg1302R primer set was developed initially for DGGE analysis and designed from only the 18S rDNA sequence of rumen ciliates (Kittelmann and Janssen, 2011). This primer set allows detection of 12 major rumen ciliate genera, which represent over 99% of total protozoal abundance across 742 samples from 32 species of ruminants (Henderson et al., 2015). The P-SSU-316F/GIC758R primer set was designed for amplicon sequencing using NGS. It was evaluated for its specificity for rumen protozoa against protozoal reference 18S rRNA gene sequences, including those of non-ruminal ciliates that were available in NCBI (Ishaq and Wright, 2014). Both primer sets allowed detection and identification of the major ruminal ciliates at the genus taxonomic rank and detected similar diversity as microscopic identification; however, rather different protozoal relative abundance resulted from the two primer sets.

Accurate quantification of rumen protozoal abundance can be difficult to achieve using qPCR or amplicon sequencing with NGS for several reasons. First, the 18S rRNA genes in rumen protozoa (as in non-rumen ciliate protozoa) reside in both micronuclei and macronuclei. In the latter, its copy number is very large, ranging from 1,010 to 6,210 copies per cell of a mixed protozoal community (Sylvester et al., 2005). In general, small entodiniomorphids have fewer 18S rRNA gene copies per cell than larger cells (Sylvester et al., 2009). Therefore, the absolute abundance (copies of 18S rRNA genes per unit of weight or volume of samples) and relative abundance (% of total protozoal sequences) of small entodinia can be underestimated by qPCR and amplicon sequencing, respectively, whereas those with a large cell, such as *Polyplastron* and *Epidinium*, are overestimated (Ishaq and Wright, 2014; Kittelmann et al., 2015). As such, 18S rRNA gene-targeting qPCR probably does not allow accurate quantification of protozoal abundance *per se* but might be associated more positively with activity if we can assume that larger cells have greater activity on a cell basis (Wenner et al., 2018). Kittelmann et al. (2015) showed that microscopic counting was more accurate than high-throughput sequencing using primer set RP841F/Reg1302R in determining protozoal abundance. Equally challenging is the determination of protozoal biomass using qPCR or amplicon sequencing with NGS because of the unknown copy numbers of 18S rRNA genes per cell for all the genera and species of rumen protozoa. Regulation of copy number of 18S rRNA genes in ruminal ciliates also is poorly understood (other than the obvious replication before cytokinesis).

### Primer Coverage and Specificity for Protozoal Community Analysis

Individual protozoal genera and species contribute differently to the overall rumen function both qualitatively and quantitatively. Thus, quantification of individual ciliate genera, if not species, is needed to assess their associations with dietary interventions. Hereto, at least 15 genera, all of which belong to the subclass *Trichostomatia*, of rumen ciliates have been identified based on 18S rRNA genes (Table 1). Because the 18S rRNA gene is highly conserved among different genera of protozoa, genera- or species-specific primers have been difficult to design. Only one study reported one pair of *Entodinium*-specific and one pair of *Dasytricha ruminantium*-specific primers, which allowed quantification of these two taxa (Skillman et al., 2006). Based on our *in silico* evaluation using TestPrime 1.0^1^ as documented by Klindworth et al. (2013), both primer sets are specific for their targets (as assumed for up to one mismatch), but the *Entodinium-*specific primer set could only achieve 51.7% coverage (data not shown). Clearly, further research is needed to improve upon the nearly 50% of the missing *Entodinium* spp. sequences and to verify specificity with one or even no mismatch allowed. To overcome the limitation of microscopic counting and morphologic identification (described above), specific primers for at least the major genera of rumen protozoa need to be developed and adequately validated; until then, we recommend for cell counting to be continued.

**TABLE 1:** Evaluation of 18S rRNA published gene-targeting primer pairs using the SILVA database 18S rRNA genes of rumen protozoa^1^.

	RP841F/Reg1302R (Kittelmann et al., 2015)			P-SSU-316F/GIC758R (Ishaq and Wright, 2014)			GIC1080F/GIC1578R (Ishaq and Wright, 2014)			P-SSU-54F/P-SSU-1747R (Sylvester et al., 2004)			P.324f/P.1747r_2 (Zhang et al., 2015)		
Genus targeted	Eligible target sequences^2^	Coverage (%)^3^	Specificity (%)^4^	Eligible target sequences	Coverage (%)	Specificity (%)	Eligible target sequences	Coverage (%)	Specificity (%)	Eligible target sequences	Coverage (%)	Specificity (%)	Eligible target sequences	Coverage (%)	Specificity (%)
*Charonina*	3	0	0	2	50.0	100	3	33.3	99.9	2	100	99.9	3	33.3	100
*Dasytricha*	22	72.7	99.9	12	100	100	20	75.0	99.9	5	100	99.9	16	37.5	100
*Diplodinium*	34	100	99.9	17	88.2	100	33	54.5	99.9	15	100	99.9	0	0	NA
*Diploplastron*	2	100	99.9	1	100	100	2	100	99.9	1	100	99.9	0	0	NA
*Enoploplastron*	1	100	99.9	1	100	100	1	100	99.9	1	100	99.9	0	0	NA
*Entodinium*	201	89.1	100	38	92.1	100	196	80.1	99.9	16	100	99.9	175	20.0	100
*Eodinium*	3	100	99.9	3	100	100	0	0	NA	0	0	NA	0	0	NA
*Epidinium*	3	100	99.9	2	100	100	3	100	99.9	2	100	100	0	0	NA
*Eremoplastron*	5	40.0	99.9	5	80.0	100	5	80.0	99.9	4	75.0	99.9	0	0	NA
*Eudiplodinium*	36	100	99.9	34	100	100	36	33.3	99.9	33	97.0	100	0	0	NA
*Isotricha*	8	0	0	6	100	100	8	75.0	99.9	6	100	99.9	0	0	NA
*Metadinium*	6	100	99.9	4	100	100	6	66.7	99.9	4	100	99.9	0	0	NA
*Ophryoscolex*	43	93.0	99.9	31	96.8	100	43	41.9	99.9	30	100	100	46	2.2	100
*Ostracodinium*	5	100	99.9	3	100	100	5	100	99.9	3	100	99.9	0	0	NA
*Polyplastron*	27	88.9	99.9	21	90.5	100	27	22.2	99.9	20	95.0	99.9	30	6.7	100
uncultured	17	94.1	99.9	0	0	NA	17	94.1	99.9	0	0	NA	17	64.7	100
Total	416	88.2^5^	99.9	180	94.4	100	405	66.2	99.9	142	97.9	99.9	287	19.5	100

*^1^TestPrime 1.0 was used to evaluate the primer pairs (Klindworth et al., 2013).^2^Eligible target seqs = number of target sequences with corresponding regions of the primer pairs.^3^Coverage (%) = (number of sequences matched/number of eligible target sequences) × 100. Total coverage mean (bottom row of data) among all genera is weighted for the numbers of sequences within each genus.^4^Specificity (%) = 100 - (outgroup matched sequences/outgroup matchable sequences) × 100. NA = not available because it could be calculated only when at least one sequence was matched. NA values were excluded from the total mean.^5^With one mismatch allowed (and not compromising specificity), coverage of this primer pair equals 95.9%.*

To make this section more useful to some readers who need to choose a primer set in analyzing rumen protozoa, we evaluated the primers that have been used commonly with respect to their coverage and specificity using *in silico* evaluation. Briefly, using TestPrime 1.0, we compared the sequences of each primer set against the recent SILVA non-redundant SSU reference dataset (SSU r132), which contains over 400 reference 18S rRNA gene sequences representing 15 genera and 29 species of protozoa, including *C. ventriculi* (Kittelmann et al., 2015) and *Eodinium posterovesiculatum* (Cedrola et al., 2017), within subclass *Trichostomatia*, which covers all rumen ciliates. Coverage and specificity of each primer set were calculated as below (Raymann et al., 2017):

Coverage(%)=(numberofsequencesmatched÷thenumberofeligibletargetsequences)×100

Specificity(%)=100-[(numberofoutgroupmatchedsequences÷numberofoutgroupmatchablesequences)×100]

Table 1 summarizes the evaluation results of the primer sets. All those tested primer sets are specific for ciliates within the phylum *Ciliophora*, except the GIC1080F/GIC1578R set, which matched one algal sequence. However, none of the primer sets evaluated could achieve complete inclusive coverage (i.e., 100%), thus probably leaving some genera undetected. Among the three primer sets that can generate amplicons with a length suitable for qPCR and NGS (RP841F/Reg1302R, P-SSU-316F/GIC758R, and GIC1080F/GIC1578R), the P-SSU-316F/GIC758R primer pair achieved the highest average coverage (94.4% total coverage). With one mismatch allowed for the primer evaluation, RP841F/Reg1302R achieved 95.9% total coverage with all 15 rumen protozoal genera detected (data not shown). Of the two primer sets that can generate nearly full-length amplicons of protozoal 18S rRNA genes, the primer set P-SSU-54F/P-SSU-1747R achieved a coverage totaling 97.9%. Until new specific primers with improved coverage are developed, we recommend for these three primer sets to be used. Two primer sets, RP841F/Reg1302R and P-SSU-316F/GIC758R, are most suitable for qPCR and preparation of amplicon libraries of 18S rRNA genes of rumen microbiota, whereas P-SSU-54F/P-SSU-1747R is more suitable for generating nearly full-length amplicons for sequencing using the third generation sequencing (TGS) technologies, such as PacBio^2^.

The NGS technologies have enabled comprehensive analysis of diverse and complex microbial communities since 2004 (Handelsman, 2004; Jovel et al., 2016). They are the primary technologies used in metagenomic studies of prokaryotic microbiota (Jovel et al., 2016), including rumen microbiota (Denman et al., 2018). Building on previous functional information based largely on monocultures (Williams and Coleman, 1992) and biochemical analyses (Béra-Maillet et al., 2005), metatranscriptomics techniques have emphasized the importance of protozoa to ruminal fiber degradation (Dai et al., 2015; Comtet-Marre et al., 2017), thus more closely relating protozoal functions to production situations. Gene sequencing approaches are being used more routinely to assess protozoal community structure *in vivo*. The first study that used NGS in sequencing rumen protozoal 18S rRNA genes to analyze the protozoal, prokaryotic, and fungal microbiota was reported by Kittelmann et al. (2013) after which Ishaq and Wright (2014) designed and evaluated the primers for specific analysis of rumen protozoal communities using NGS. These primers have enabled comprehensive and efficient analysis of the rumen protozoal populations in the rumen microbial community, including variations caused by different diets (Ishaq et al., 2017; Zhang et al., 2017; Bainbridge et al., 2018), breed and lactation stage in dairy cattle (Cersosimo et al., 2016), and geographical regions among moose samples (Ishaq et al., 2015).

Most of the 18S rRNA gene sequences of rumen protozoa archived in public databases are partial sequences. Improvement of current primers and design of new primers with desirable specificity and coverage require more reference sequences with accurate taxonomic identification, ideally at the species or OTU (operational taxonomic unit; currently at 97% similarity) rank. Reference sequences need to be full- or nearly full-length to allow proper primer design and *in silico* evaluation. None of the NGS platforms in use can sequence the full-length 18S rRNA gene of protozoa. However, the TGS techniques, such as PacBio RS II, produces sequence reads averaging over 10,000 base pairs. TGS can sequence the entire rRNA operon, which is composed of one 18S rRNA gene, one internal transcribed spacer (ITS1), one 5.8S rRNA gene, one ITS2, and one 28S rRNA gene. All but the 5.8S rRNA gene are useful phylogenetic markers. Full-length sequences of the entire operon allow not only the design of universal primers specific for rumen protozoa, but also the design of genera- and species-specific primers. Indeed, ITS1, ITS2, and the 28S rRNA gene are better markers for ciliate identification (Abraham et al., 2019). Species-specific primers would allow researchers to reliably identify rumen protozoal species of interest using minimal expertise based on morphological features and to determine dietary shifts in the relative abundance of individual rumen protozoal species.

Although many researchers have attempted to develop protozoal axenic cultures (having no influence by prokaryotes, which contrasts with protozoal monocultures), no laboratory maintainable axenic cultures have been established (Park et al., 2017). The lack of an axenic culture of any ruminal ciliate species has hindered understanding of their metabolism, physiology, and ecology; thus, their actual roles in the rumen could only be inferred from indirect evidence (Coleman, 1962; Hino and Kametaka, 1977; Onodera and Henderson, 1980; Fondevila and Dehority, 2001b). Genomics and transcriptomics, empowered by NGS and TGS technologies, would be enabling approaches to help gain new insights into the fundamental biological features of rumen protozoa. Genomic (Park et al., 2018) and transcriptomic (Wang et al., 2020) studies of *En. caudatum* are providing starting points for future studies investigating roles of prokaryotes or interactions such as in defaunation studies (discussed previously).

### Protozoa-Associated Bacteria

Rumen protozoa interact intensively with other members of the rumen microbiota. Predation, symbiosis, and cross-feeding are among the major interactions between protozoa and other members of the rumen microbiota. These interactions can shape the rumen microbiota (diversity, composition, and dynamics) and its functions and therefore are key to advancing our understanding of rumen microbial ecosystem. The most obvious and well-documented interaction is predation of bacteria (Diaz et al., 2014; Newbold et al., 2015; Rossi et al., 2019). Although still debatable if rumen ciliates do select their preys (Dehority, 2003; Belanche et al., 2011), the enriched intracellular *Proteobacteria* populations in other free-living ciliates in marine or freshwater environments might correlate to the symbiotic relationship between bacteria and host ciliates (Görtz and Brigge, 1998; Gong et al., 2016). Selective predation of rumen bacteria by rumen protozoa, at least *En. caudatum*, is likely (Park and Yu, 2018b); however, more research is needed to determine the prey selections by individual species of rumen protozoa and how prey selectivity is achieved.

Live bacteria are required as sources of essential nutrients that cannot be produced or obtained from other sources (Fondevila and Dehority, 2001b; Park et al., 2017). In addition to endosymbionts, live bacteria (and possibly archaea) might be sources of prey or symbionts for recurrent recruitment. External bacteria consumed by or harvested with protozoa are difficult to distinguish from endosymbiont prokaryotes. As generated from washed single rumen ciliate cells, the 16S rRNA gene amplicons of protozoa-associated prokaryotic community seem to have a composition that is different from that of free-living prokaryotes of the rumen (Irbis and Ushida, 2004; Levy and Jami, 2018; Park and Yu, 2018b). Some taxa (ranging from genus to order) were exclusively associated with rumen ciliate cells (Table 2). However, the detection of “protozoa-associated” species of bacteria probably reflects both the selective predation and presence of symbionts (Gong et al., 2016; Park and Yu, 2018b). In contrast with typical ruminal bacteria (primarily in the phyla *Firmicutes* and *Bacteroidetes*), some species of *Proteobacteria* (mostly within *α-* and *γ-Proteobacteria* classes) have been exclusively found inside various ciliates (Gong et al., 2016). A few of them, such as *Polynucleobacter* spp., are obligate endosymbionts in several species of ciliates (Soldo, 1987; Görtz and Brigge, 1998). Therefore, some of the *α-* and *γ-Proteobacteria* that were associated with rumen protozoa appear to be true endosymbionts. No archaeal sequences were detected exclusively in protozoa (i.e., they were also detected in the free-living pool) in the study of Park and Yu (2018b). Although archaea can interact intimately with protozoa, some methanogens interact extracellularly with ciliates, and some intracellular methanogens might be continually replaced by ingestion rather than being true endosymbionts (Ushida, 2010). Future research can further identify specific prokaryotes and reveal their potential mutualistic interactions with rumen ciliates. As suggested previously (Park and Yu, 2018b), single-cell microbiomics coupled with controlled starvation of protozoa cells is one effective approach to distinguish continually ingested prokaryotes from endosymbionts.

**TABLE 2:** Relative sequence abundance of major bacterial taxa exclusively found in single cells of freshly isolated rumen ciliates^1^.

Phylum	Ciliate genera							
Lowest taxa assigned^2^	*Dasytricha*	*Diplodinium*	*Diploplastron*	*Entodinium*	*Epidinium*	*Isotricha*	*Ophryoscolex*	*Polyplastron*
*Acidobacteria*	1.72	1.86	1.48	1.38	1.03	0.92	0.98	1.68
*Actinobacteria*								
o_*Acidimicrobiales*	3.41	3.58	3.49	3.16	1.98	2.84	4.12	1.66
*Bacteroidetes*								
f_*Chitinophagaceae*	4.90	3.94	4.04	4.25	4.05	4.84	5.10	2.15
g_*Sediminibacterium*	4.78	3.63	3.62	3.53	3.54	4.22	4.70	2.14
*Proteobacteria*								
o_*Ellin329*	2.34	1.57	1.00	1.84	2.86	1.20	1.71	2.20
o_*Rhizobiales*	1.36	2.23	1.37	1.06	1.36	1.65	1.74	1.71
f_*Bradyrhizobiaceae*	1.09	1.43	0.91	0.85	0.69	1.46	1.29	1.45
f_*Rhodospirillaceae*	2.88	3.60	1.26	1.90	2.27	2.08	1.88	1.60
g_*Limnobacter*	1.51	2.51	2.06	1.01	1.77	2.84	1.68	1.71
c_*ε-Proteobacteria*	1.88	1.37	1.96	2.94	1.43	0.33	0.79	1.55
o_*Xanthomonadales*	4.36	3.52	2.26	5.55	3.66	3.11	3.81	1.65
f_*Sinobacteraceae*	4.25	2.56	1.92	4.77	3.10	2.97	3.64	1.53
g_*Nevskia*	2.74	1.56	1.36	2.29	1.41	1.55	1.49	1.06

*^1^Relative abundance (% of total prokaryotic sequences) retrieved from isolated single cells of rumen ciliates extracted from fresh rumen fluid. No archaeal sequences were recovered that were exclusive to protozoa. Excerpted from Park and Yu (2018b). ^2^The lowest classified taxa are class (c), order (o), family (f), or genus (g).*

## Conclusion

From monocultures to *in vivo* approaches, our colleague Burk Dehority was the consummate gentleman scholar. He always considered the most parsimonious approach to test a hypothesis, yet he always considered the “because they can” interpretation of results (avoiding teleology) within the larger context of advancing our ability to better define roles for ruminal protozoa with respect to positives and negatives both qualitatively and quantitatively. The topics discussed herein often stem from many discussions, particularly those traveling back from meetings that stimulated new questions, techniques, and approaches to science. This review was written in his honor “because we could.”

## Author Contributions

JF initiated the review and wrote a first draft. ZY, TP, and JP made contributions in content and added to the draft.

## Conflict of Interest

The authors declare that the research was conducted in the absence of any commercial or financial relationships that could be construed as a potential conflict of interest.

## Footnotes

1 https://www.arb-silva.de/search/testprime/

2 https://www.pacb.com/
